# Screening for Complex Diseases and Personalized Health Care

**DOI:** 10.1155/2015/135931

**Published:** 2015-01-14

**Authors:** Stefania Boccia, Paolo Boffetta, Paolo Villari

**Affiliations:** ^1^Section of Hygiene, Institute of Public Health, Università Cattolica del Sacro Cuore, Largo Francesco Vito 1, 00168 Rome, Italy; ^2^IRCCS San Raffaele Pisana, Via della Pisana 235, 00163 Rome, Italy; ^3^The Tisch Cancer Institute and Institute for Translational Epidemiology, Icahn School of Medicine at Mount Sinai, New York, NY, USA; ^4^Department of Public Health and Infectious Diseases, Sapienza University of Rome, Piazzale Aldo Moro 5, 00185 Rome, Italy

The extraordinary accomplishment of decoding the human genome, together with the technological advancement of the later part of the 20th century, has strong implications for the health care, as it provides the opportunity to transform its delivery health from a disease-focused model to one that is personalized, predictive, proactive, precise, and patient-centred. Personalized health care can be broadly defined as a customization of the medical provision that accommodates individual differences in all stages in the process, from prevention, to diagnosis and treatment, to posttreatment follow-up. Despite the fast moving human genome discoveries in a wide proportion of diseases having large public health impact, however, the promise of personalized healthcare has far lagged behind due the complexity involved [1]. In addition, besides the pharmacogenomics field, the evidence on the extent to which genomic information has provided measurable population health benefits and actionable intelligence to citizens is still limited.

This special issue includes five contributions spanning from genetic association studies to the awareness of citizens on cervical cancer risk factors and screening programs and physician knowledge on genetic tests for diagnosis of hereditary cancer.

The first set of papers is represented by three genetic association studies on chronic diseases. The paper of F. Coppedè et al. reported on the absence of association between the common c.80G>A polymorphism (rs1051266) of the gene coding for the reduced folate carrier (*RFC*-1 ge) and Alzheimer's disease in an Italian case-control studies. Similarly, despite the potential for a biological significance of *TNFA *−308G>A polymorphism on the risk of obesity, M. Barchitta et al. reported the absence of gene-Mediterranean diet interaction on the risk of overweight/obesity among Italian women. A poor adherence to the Mediterranean diet, however, was associated with educational level (less common among those less educated) and younger ages. Lastly, the paper of S. Boccia et al. investigated the effect of* CYP*,* GST*, and* SULT* polymorphisms and their interaction with smoking on the risk of hepatocellular carcinoma. Results show that* CYP2E1* ^∗^ *5B* and* CYP2E1* ^∗^ *6* polymorphisms have a favorable effect on the development of HCC, while polymorphisms of* GSTT1* and* SULT1A1* might play role in increasing the susceptibility among smokers.

The paper of C. D. Vito et al. investigated the knowledge of cervical cancer risk factors and the predictors of adherence to cervical cancer screening in relation to mass media campaigns in a large study conducted in Italy. Results show that higher educational level, being not married, and living in urban areas were the main independent characteristics associated with a higher level of knowledge of cervical cancer etiology. During the campaign period women had the Pap test more frequently as a consequence of the mass media campaign, strengthening the evidence of the usefulness of media campaigns via local television to foster cervical screening compliance.

Lastly, the paper of N. Panic et al. assessed the knowledge and attitudes on genetic tests for breast and colorectal cancer of young medical residents of postgraduates schools. The study represents a follow-up of a larger study of a sample of Italian physicians [2]. Results show that knowledge on hereditary breast cancer genetic tests was high; for colorectal cancer it was largely insufficient. Knowledge on tests was higher among residents who attended course on cancer genetic testing during graduate training. More than 70% asked for the additional training on the genetic tests for cancer during the specialization school.

*Stefania Boccia* *Paolo Boffetta* *Paolo Villari*
